# Imeglimin mitigates the accumulation of dysfunctional mitochondria to restore insulin secretion and suppress apoptosis of pancreatic β-cells from *db/db* mice

**DOI:** 10.1038/s41598-024-56769-w

**Published:** 2024-03-14

**Authors:** Kyota Aoyagi, Chiyono Nishiwaki, Yoko Nakamichi, Shun-ichi Yamashita, Tomotake Kanki, Mica Ohara-Imaizumi

**Affiliations:** 1 Department of Cellular Biochemistry, Kyorin University School of Medicine, Mitaka, Tokyo 181-8611 Japan; 2 Department of Cellular Physiology, Niigata University Graduate School of Medical and Dental Sciences, Niigata, 951-8510 Japan

**Keywords:** Endocrine system and metabolic diseases, Autophagy

## Abstract

Mitochondrial dysfunction in pancreatic β-cells leads to impaired glucose-stimulated insulin secretion (GSIS) and type 2 diabetes (T2D), highlighting the importance of autophagic elimination of dysfunctional mitochondria (mitophagy) in mitochondrial quality control (mQC). Imeglimin, a new oral anti-diabetic drug that improves hyperglycemia and GSIS, may enhance mitochondrial activity. However, chronic imeglimin treatment’s effects on mQC in diabetic β-cells are unknown. Here, we compared imeglimin, structurally similar anti-diabetic drug metformin, and insulin for their effects on clearance of dysfunctional mitochondria through mitophagy in pancreatic β-cells from diabetic model *db/db* mice and mitophagy reporter (CMMR) mice. Pancreatic islets from *db/db* mice showed aberrant accumulation of dysfunctional mitochondria and excessive production of reactive oxygen species (ROS) along with markedly elevated mitophagy, suggesting that the generation of dysfunctional mitochondria overwhelmed the mitophagic capacity in *db/db* β-cells. Treatment with imeglimin or insulin, but not metformin, reduced ROS production and the numbers of dysfunctional mitochondria, and normalized mitophagic activity in *db/db* β-cells. Concomitantly, imeglimin and insulin, but not metformin, restored the secreted insulin level and reduced β-cell apoptosis in *db/db* mice. In conclusion, imeglimin mitigated accumulation of dysfunctional mitochondria through mitophagy in diabetic mice, and may contribute to preserving β-cell function and effective glycemic control in T2D.

## Introduction

In response to elevated blood glucose levels, pancreatic β-cells exhibit glucose uptake and metabolism to generate ATP, mainly through mitochondrial respiration. The increase in cytosolic ATP triggers depolarization of the plasma membrane, influx of extracellular Ca^2+^, and insulin secretion to reduce blood glucose levels^1,2^. Consistent with the central role of mitochondria in coupling glucose metabolism to insulin secretion, mitochondrial abnormalities in pancreatic β-cells have been shown to cause defective insulin secretion and hyperglycemia. For example, mutations in mitochondrial DNA, which disrupt mitochondrial function, were found in patients with hereditary diabetes^3^, and mice with genetically induced mitochondrial dysfunction showed impaired insulin secretion and hyperglycemia^4^. Meanwhile, morphologically abnormal mitochondria, a feature of potentially dysfunctional mitochondria, have been observed in pancreatic β-cells in rodent models of obesity and in patients with type 2 diabetes (T2D)^5–7^. Additionally, recent studies have revealed that loss of mitochondrial quality control (mQC) in pancreatic β-cells is involved in the onset of T2D. Loss of mQC results in the accumulation of dysfunctional mitochondria, which leads to excessive production of reactive oxygen species (ROS) and cell death. Thus, elimination of dysfunctional mitochondria is essential to prevent β-cell dysfunction^2,8^. Cells maintain mQC by eliminating dysfunctional mitochondria through mitochondrial autophagy (hereafter referred to as mitophagy). Furthermore, studies of mice lacking proteins required for autophagy/mitophagy regulation showed the accumulation of dysfunctional mitochondria, impairment of glucose-stimulated insulin secretion (GSIS) and hyperglycemia^9–11^. Thus, restoration of mitochondrial function and maintenance of mQC in β-cells could be beneficial for the treatment of T2D patients.

Imeglimin is a new oral anti-diabetic drug that is structurally related to metformin^12^, a medication widely used to treat T2D. Imeglimin has been shown to reduce blood glucose levels in diabetic model rodents^13–17^ and T2D patients^18,19^ via the following mechanisms. Studies demonstrated that imeglimin’s euglycemic effect is exerted by improving the uptake of blood glucose by insulin in skeletal muscle and by inhibiting glycogenesis in the liver^13,14^. Additionally, in pancreatic β-cells, imeglimin was shown to potentiate GSIS^15,20,21^, probably by improving mitochondrial function. Specifically, Hallakou-Bozec and colleagues reported that imeglimin upregulated nicotinamide phosphoribosyltransferase (NAMPT) expression to increase the content of cellular nicotinamide adenine dinucleotide (NAD^+^), an essential co-factor for various cellular metabolic reactions, as well as ATP in islets isolated from diabetic model Goto-Kakizaki (GK) rats^22^. Furthermore, recent papers showed that imeglimin treatment also improved mitochondrial morphology and suppressed apoptosis in β-cells in diabetic model *db/db* mice and GK rats^15–17,23^. Thus, imeglimin may improve hyperglycemia by enhancing insulin secretion and preserving β-cell mass, likely via the maintenance of mQC in pancreatic β-cells. However, it remains unclear whether imeglimin affects mQC in diabetic β-cells.

In the present study, we examined the effects of imeglimin on the maintenance of mQC from the perspective of mitophagy in pancreatic β-cells from *db/db* mice. We also compared the effects of imeglimin with the structurally similar metformin and with insulin, which maintains functional β-cells in T2D patients^24,25^ and has been shown to reduce dysfunctional mitochondria via mitophagy in β-cells in high-fat diet (HFD)-fed mice^26^. Our results suggest that imeglimin mitigates the accumulation of dysfunctional mitochondria in β-cells from *db/db* mice, leading to an increase in the amount of secreted insulin, suppression of apoptotic β-cell death and amelioration of hyperglycemia.

## Results

### Effects of imeglimin, metformin and insulin on glycemic control in *db/db* mice

**Figure 1:**
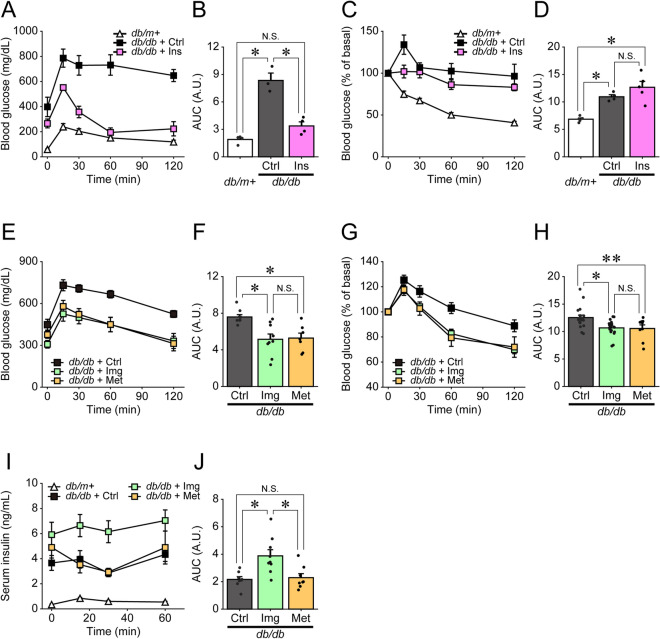
Effects of imeglimin, metformin and insulin on glycemic control in *db/db* mice. (**A**,**B**) Oral glucose tolerance tests (0.8 g glucose/kg body weight) (**A**) and AUC analysis (**B**) performed on *db/m* + (n = 4) and *db/db* mice treated with saline (*db/db* + Ctrl; n = 3) or insulin (*db/db* + Ins; n = 4); *p < 0.01 by Tukey’s honestly significant difference (HSD) test. (**C**,**D**) Insulin tolerance tests (1.5 U/kg body weight) (**C**) and AUC analysis (**D**) performed on *db/m* + (n = 5) and *db/db* + *Ctrl;* (n = 4) or *db/db* + Ins (n = 5) mice; *p < 0.01 by Welch’s t-test, with p values adjusted by Holm’s method. (**E**,**F**) Oral glucose tolerance tests (0.8 g glucose/kg body weight) (**E**) and AUC analysis (**F**) performed on *db/db* mice treated with saline (Ctrl; n = 9), imeglimin (Img; n = 9) or metformin (Met; n = 7); *p < 0.01 by Tukey’s HSD test. (**G**,**H**) Insulin tolerance tests (1.5 U/kg body weight) (**G**) and AUC analysis (**H**) performed on *db/db* mice treated with saline Ctrl (n = 19), Img (n = 19) or Met (n = 9); *p < 0.01 and **p < 0.03 by Tukey’s HSD test. (**I**,**J**) Serum insulin measurements (**I**) and AUC analysis (**J**) of blood samples from *db/m* + mice (n = 12) or *db/db* mice treated with saline Ctrl (n = 8), Img (n = 9) or Met (n = 8). Serum samples were recovered from mice subjected to OGTT; *p < 0.01 by Tukey’s HSD test.

We first examined the effects of imeglimin, metformin and insulin on glycemic control in *db/db* mice. Oral glucose tolerance test (OGTT) and insulin tolerance test (ITT) were performed following 6 weeks of treatment with orally administered imeglimin or metformin, or chronically administered insulin using an osmotic minipump. Insulin treatment reduced blood glucose levels (Fig. 1A,B), but did not affect insulin sensitivity (Fig. 1C,D), whereas both imeglimin and metformin improved blood glucose levels and insulin sensitivity (Fig. 1E–H). However, the amount of serum insulin was increased in imeglimin-treated, but not metformin-treated, *db/db* mice (Fig. 1I,J), suggesting that imeglimin and metformin ameliorate hyperglycemia through different mechanisms in *db/db* mice. These results indicated that imeglimin was effective at improving pancreatic β-cell dysfunction as well as insulin sensitivity in some peripheral tissues.

### Imeglimin and insulin, but not metformin, reduce dysfunctional mitochondria in β-cells from *db/db* mice

**Figure 2:**
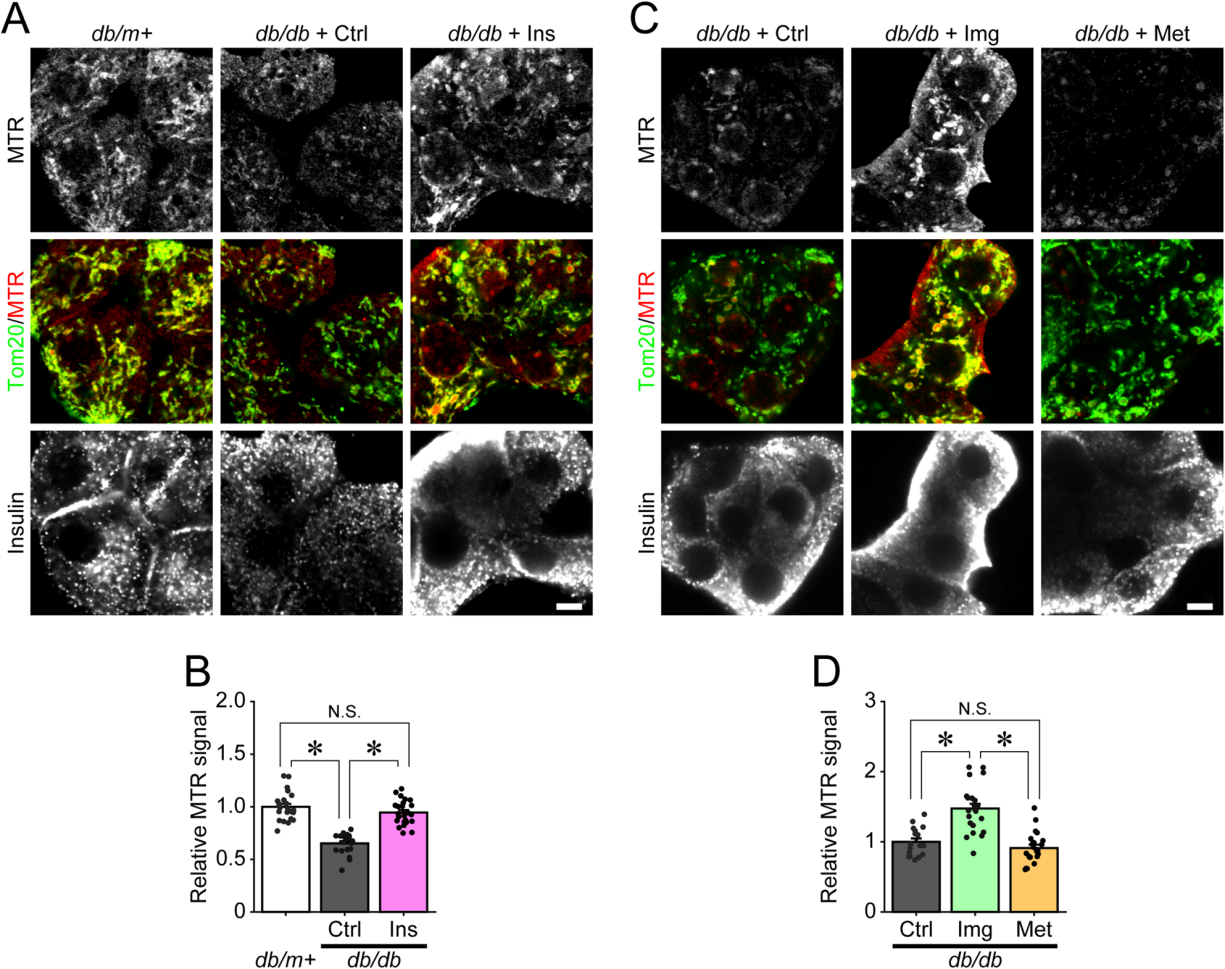
Imeglimin and insulin, but not metformin, reduce dysfunctional mitochondria in pancreatic β-cells. (**A**,**C**) Pancreatic β-cells were cultured from *db/m* + mice or *db/db* mice administered saline (Ctrl), insulin (Ins), imeglimin (Img) or metformin (Met) and stained with 0.5 μM MTR (red) for 30 min, followed by immunostaining for insulin (gray) and Tom20 (green). (**B**) Relative MTR/Tom20 signal ratios of the groups shown in (**A**); n = 24, 20 and 24 images for *db/m* + , *db/db* + Ctrl and *db/db* + Ins, respectively; *p < 0.01 by Tukey’s HSD test. (**D**) Relative MTR/Tom20 signal ratios of the groups shown in (**C**); n = 17, 24 and 22 images for *db/db* + Ctrl, *db/db* + Img and *db/db* + Met, respectively; *p < 0.01 by Tukey’s HSD test. Scale bars 5 µm.

Impaired mitochondrial function and morphologically abnormal mitochondria have been detected in pancreatic β-cells from diabetic model rodents and T2D patients^5–7^. Additionally, our previous study showed that dysfunctional mitochondria accumulated in β-cells from HFD-fed mice^26^. Because dysfunctional mitochondria are detrimental to cellular homeostasis and lead to β-cell dysfunction^2,8^, we hypothesized that imeglimin may reduce the numbers of dysfunctional mitochondria in β-cells from *db/db* mice. To test this hypothesis, we cultured β-cells from control-treated or drug-treated *db/db* mice and visualized functional mitochondria using Mitotracker Orange CM-H_2_TMRos (MTR), a fluorescent dye that stains functional mitochondria. Concomitantly, we immunostained the cells for translocase of the outer membrane 20 (Tom20) to identify all mitochondria, and then evaluated the signal intensity of MTR in the regions stained with Tom20. Compared with that in non-diabetic *db/m* + control mice, the signal intensity of MTR was markedly decreased in β-cells cultured from *db/db* mice, suggesting a reduction in functional mitochondria with the aberrant accumulation of dysfunctional mitochondria in *db/db* β-cells (Fig. 2A,B). The signal intensity of MTR was restored in β-cells cultured from insulin-treated *db/db* mice to the level of those from *db/m* + control mice (Fig. 2A,B). Meanwhile, the signal intensity of MTR was significantly elevated in β-cells cultured from imeglimin-treated, but not metformin-treated, *db/db* mice (Fig. 2C,D).

**Figure 3:**
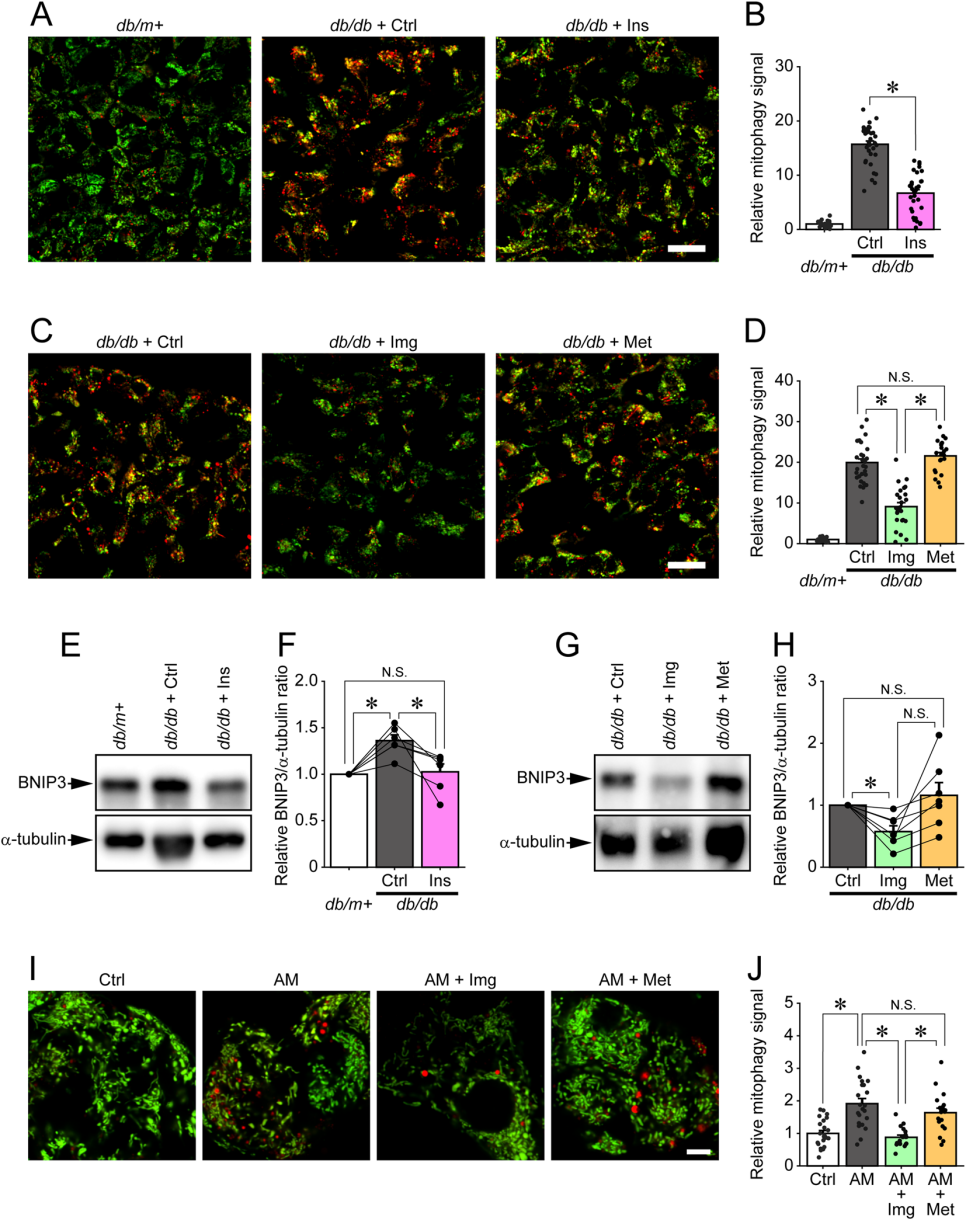
Imeglimin and insulin, but not metformin, suppress mitophagic activity in pancreatic β-cells in vivo. (**A**,**B**) Representative images (**A**) and relative numbers of mitophagic signals (**B**) of pancreatic sections from *CMMR;db/m* + (*db/m* + ; n = 30 islets) and *CMMR;db/db* mice administered saline (*db/db* + Ctrl; n = 35 islets) or insulin (*db/db* + Ins; n = 33 islets); *p < 0.01 by Welch’s t-test, with p values adjusted by Holm’s method. (**C**,**D**) Representative images (**C**) and relative numbers of mitophagic signals (**D**) of pancreatic sections from *CMMR;db/db* mice administered saline (*db/db* + Ctrl; n = 25 islets), imeglimin (*db/db* + Img; n = 30 islets) or metformin (*db/db* + Met; n = 22 islets); *p < 0.01 by Welch’s t-test, with p values adjusted by Holm’s method. Green signals indicate EGFP; red signals indicate mCherry. (**E**) Islets isolated from *db/m* + mice or *db/db* mice treated with saline (*db/db* + Ctrl) or insulin (*db/db* + Ins) were subjected to immunoblotting for BNIP3 and α-tubulin. Original membrane and immunoblot images were shown in Supplemental Fig. S1. (**F**) Relative amounts of BNIP3 in (**E**) (n = 6 for each group). *p < 0.05 by paired t-test, with p values adjusted by Holm’s method. (**G**) Islets isolated from *db/db* mice administered saline (*db/db* + Ctrl), imeglimin (*db/db* + Img) or metformin (*db/db* + Met) were subjected to immunoblotting for BNIP3 and α-tubulin. Original membrane and immunoblot images were shown in Supplemental Fig. S1. (**H**) Relative amounts of BNIP3 in (**G**) (n = 7 for each group). *p < 0.05 by paired t-test, with p values adjusted by Holm’s method. (**I**) Representative images of pancreatic β-cells of *CMMR*;*db/m* + mice cultured with 1 nM antimycin A (AM) together with 1 mM imeglimin (AM + Img) or 1 mM metformin (AM + Met) for 24 h. (**J**) Relative numbers of mitophagic signals in (**I**); n = 23, 22, 18 and 19 images for Ctrl, AM, AM + Img and AM + Met, respectively. *p < 0.01 by Welch’s t-test, with p values adjusted by Holm’s method. Scale bars 20 µm (**A**,**C**), 5 µm (**I**).

Mitophagy is activated by the accumulation of dysfunctional mitochondria to preserve cellular homeostasis^2,8^, and our previous study demonstrated that HFD feeding induced the accumulation of dysfunctional mitochondria and upregulated mitophagic activity in pancreatic β-cells^26^. Taken together, these observations indicated that the generation of dysfunctional mitochondria overwhelmed the degradative capacity of the mitophagy process. Given our results using MTR, we hypothesized that the mitophagic activity of β-cells from *db/db* mice would be mitigated by imeglimin and insulin treatment. To assess this possibility in vivo, we used *CMMR*^*flox/*+^*;Rip-Cre* (CMMR) mice, in which mitochondria located in cytosol emit both enhanced green fluorescent protein (EGFP) and mCherry signals, whereas mitophagy-degraded mitochondria located in lysosomes only emit the mCherry signal, especially in pancreatic β-cells^26^. *CMMR*^*flox/*+^*;Rip-Cre* mice were crossed with *db/m* + mice to generate *CMMR*^*flox/*+^*;Rip-Cre*;*db/db* (*CMMR;db/db*) mice. As shown in Fig. 3A, many mCherry-only punctate signals were observed in pancreatic β-cells from *CMMR;db/db* mice, demonstrating that mitophagic activity was upregulated, most likely by the accumulation of dysfunctional mitochondria. Insulin treatment dramatically decreased the number of mitophagy signals in *CMMR;db/db* mice (Fig. 3A,B). Correspondingly, imeglimin, but not metformin, also significantly reduced mitophagic activity (Fig. 3C,D). We also studied the effects of imeglimin, metformin and insulin on the expression levels of BNIP3, a mitophagy-related protein, in pancreatic β-cells^26,27^. As shown in Fig. 3E,F, and Supplemental Fig. S1, the amount of BNIP3 was increased in islets isolated from *db/db* mice. Consistent with the results from *CMMR*;*db/db* mice, insulin treatment significantly suppressed the expression of BNIP3. Treatment with imeglimin, but not metformin, also reduced the expression levels of BNIP3 in *db/db* mouse islets (Fig. 3G,H, and Supplemental Fig. S1). The amount of BNIP3 was higher in islets isolated from metformin-treated *db/db* mice than in those from imeglimin-treated *db/db* mice, but this did not reach statistical significance, possibly due to the high variability. To further examine whether imeglimin could facilitate the elimination of dysfunctional mitochondria in β-cells, we next induced mitochondrial dysfunction using antimycin A, an inhibitor of complex III in the mitochondrial electron transport chain^28^. As shown in Fig. 3I,J, the number of mitophagy signals was significantly increased by antimycin A treatment. Moreover, imeglimin, but not metformin, ameliorated the mitophagic activity induced by antimycin A treatment. Taken together, these results demonstrated that imeglimin and insulin, but not metformin, ameliorated the accumulation of dysfunctional mitochondria in pancreatic β-cells from *CMMR;db/db* mice.

### Imeglimin and insulin, but not metformin, reduce ROS production and oxidative stress in islet cells from *db/db* mice

**Figure 4:**
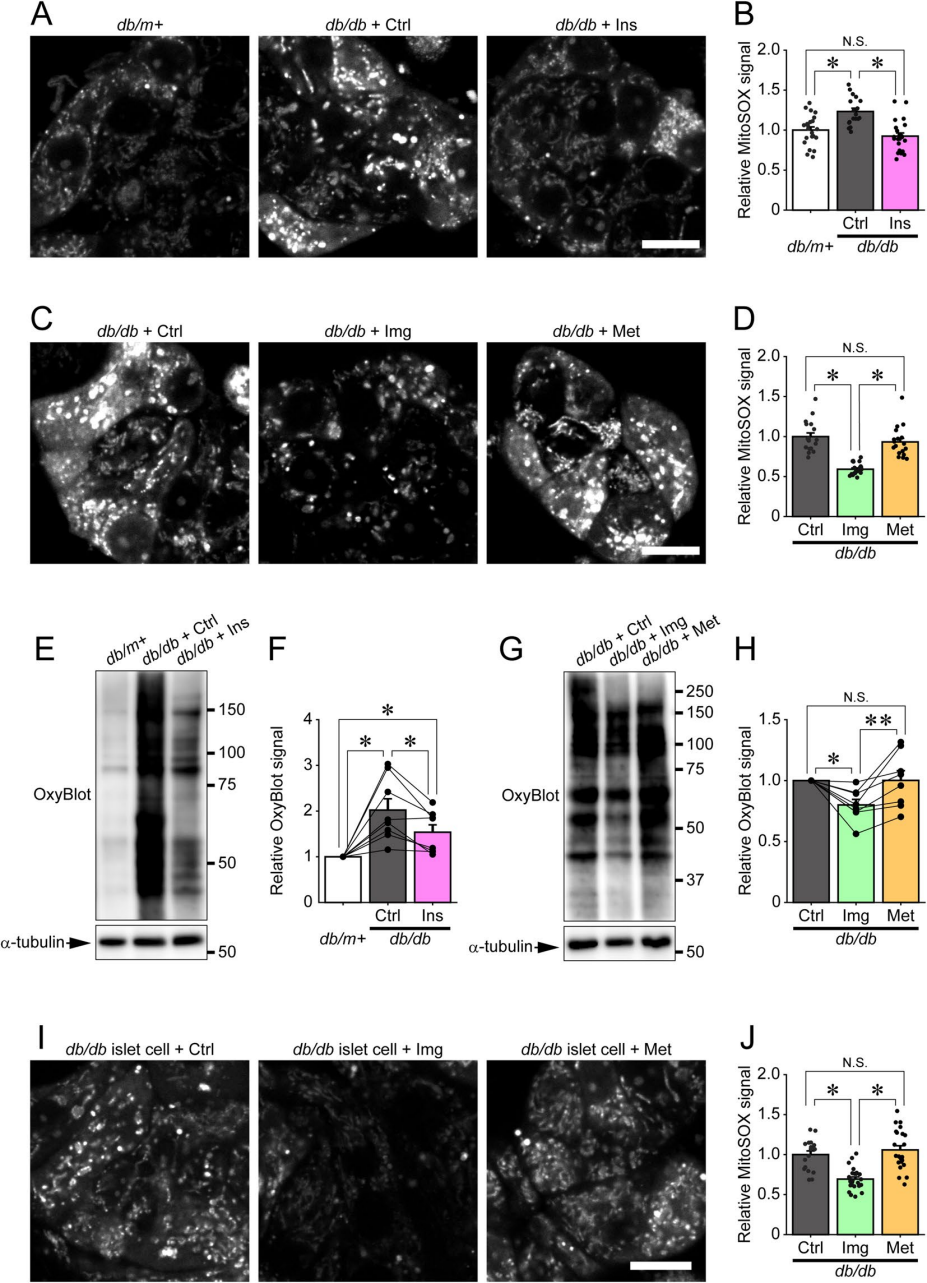
Imeglimin and insulin, but not metformin, reduce ROS generation in pancreatic β-cells. (**A**, **B**) Representative images (**A**) and relative signal intensities (**B**) of pancreatic islet cells cultured from *db/m* + (n = 21 images) or *db/db* mice administered saline (*db/db* + Ctrl; n = 18 images) or insulin (*db/db* + Ins; n = 23 images) and stained with 5 μM MitoSOX for 20 min; *p < 0.01 by Tukey’s HSD test. (**C**,**D**) Representative images (**C**) and relative signal intensities (**D**) of pancreatic islet cells cultured from *db/db* mice administered saline (*db/db* + Ctrl; n = 18 images), imeglimin (*db/db* + Img; n = 22 images) or metformin (*db/db* + Met; n = 18 images) and stained with 5 μM MitoSOX for 20 min; *p < 0.01 by Welch’s t-test, with p values adjusted by Holm’s method. (**E**,**F**) Oxidized carbonyl groups (**E**) and relative OxyBlot signal intensities (**F**) of protein extracts from isolated islets of *db/m* + mice or *db/db* mice administered saline (*db/db* + Ctrl) or insulin (*db/db* + Ins), labeled with 2,4-dinitrophenylhydrazine (DNPH) and immunoblotted with anti-2,4-dinitrophenyl (DNP) moiety antibody. OxyBlot signal intensities from 50 to 150 kDa were quantified (n = 8 for each group); *p < 0.03 by paired t-test, with p values adjusted by Holm’s method. Original membrane and immunoblot images were shown in Supplemental Figs. S2, S3. (**G**,**H**) Oxidized carbonyl groups (**G**) and relative OxyBlot signal intensities (**H**) of protein extracts from isolated islets of *db/db* mice administered saline (*db/db* + Ctrl), imeglimin (*db/db* + Img) or metformin (*db/db* + Met), labeled with DNPH and immunoblotted with anti-DNP moiety antibody; n = 8 for each group; *p < 0.01 and **p < 0.05 by paired t-test, with p values adjusted by Holm’s method. Original membrane and immunoblot images were shown in Supplemental Fig. S4. (**I**) Representative images of pancreatic islet cells of *db/db* mice cultured with 1 mM imeglimin (Img) or 1 mM metformin (Met), or without (Ctrl) for 2 days, then stained with 5 µM MitoSOX for 20 min. (**J**) Relative MitoSOX signal intensities of (I); n = 18, 25 and 23 images for Ctrl, Img and Met, respectively; *p < 0.01 by Welch’s t-test, with p values adjusted by Holm’s method. Scale bars 10 µm.

It is well established that mitochondrial dysfunction leads to excessive ROS production, which causes oxidative stress^29^. Therefore, we next examined endogenous ROS levels in β-cells from *db/db* mice. Pancreatic islet cells were cultured on coverslips from control-treated or drug-treated *db/db* mice, and stained with MitoSOX, a mitochondrial superoxide indicator. As shown in Fig. 4A,B, the MitoSOX signal intensities in islet cells cultured from control-treated *db/db* mice were significantly higher than those from *db/m* + mice. Furthermore, this increase in MitoSOX in *db/db* mice was restored to *db/m* + levels by insulin treatment. Consistently, OxyBlot analysis revealed that the amount of protein oxidized by free radicals, such as ROS, was markedly increased in *db/db* islets compared with that in *db/m* + islets, and was dramatically reduced by insulin treatment (Fig. 4E,F, and Supplemental Figs. S2–S4). Likewise, treatment with imeglimin, but not metformin, significantly reduced the MitoSOX signal intensity in cultured islet cells (Fig. 4C,D) and the OxyBlot signal intensity in isolated islets (Fig. 4G,H, and Supplemental Figs. S2–S4). We also investigated the direct effects of imeglimin on ROS production of isolated *db/db* islet cells treated in vitro. As shown in Fig. 4I,J, imeglimin, but not metformin, significantly reduced ROS production in isolated *db/db* islet cells, suggesting that imeglimin acts directly on islet cells to suppress ROS production. These results demonstrated that treatment with imeglimin or insulin, but not metformin, ameliorated ROS production and oxidative stress in islet cells from *db/db* mice.

### Imeglimin and insulin, but not metformin, increase the amount of secreted insulin and reduce apoptosis in β-cells from *db/db* mice

**Figure 5:**
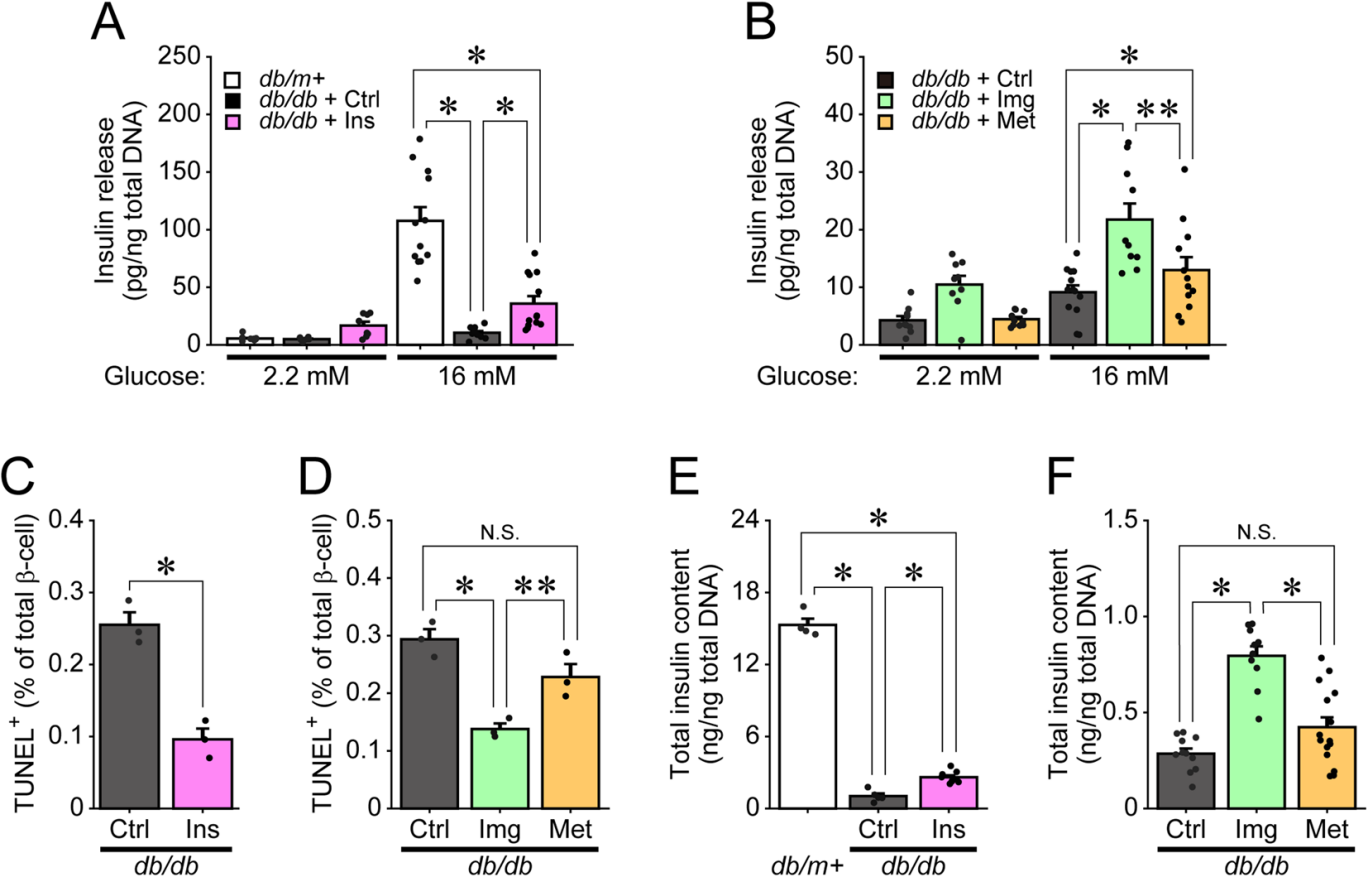
Imeglimin and insulin, but not metformin, restore GSIS and reduce apoptosis in pancreatic β-cells. (**A**) Insulin secretion in islets isolated from *db/m* + mice or *db/db* mice administered saline (*db/db* + Ctrl) or insulin (*db/db* + Ins), then stimulated with 2.2 or 16 mM glucose for 30 min (n = 12, 7 and 8 for *db/m* + , *db/db* + Ctrl and *db/db* + Ins islets under 2.2 mM glucose; n = 12, 10 and 13 for *db/m* + , *db/db* + Ctrl and *db/db* + Ins islets under 16 mM glucose, respectively); *p < 0.01 by Mann–Whitney U test, with p values adjusted by Holm’s method. (**B**) Insulin secretion in islets isolated from *db/db* mice administered saline (*db/db* + Ctrl), imeglimin (*db/db* + Img) or metformin (*db/db* + Met), then stimulated with 2.2 or 16 mM glucose for 30 min (n = 10, 9 and 10 islets from the *db/db* + Ctrl, *db/db* + Img and *db/db* + Met groups under 2.2 mM glucose; n = 13, 10 and 12 islets from the *db/db* + Ctrl, *db/db* + Img and *db/db* + Met groups under 16 mM glucose, respectively); *p < 0.01 and **p < 0.05 by Tukey’s HSD test. (**C**,**D**) Results of TUNEL staining followed by immunostaining for insulin in pancreatic sections from *db/db* mice administered Ctrl, Ins, Img or Met. The numbers of TUNEL-positive β-cells were counted (n = 3 for each group); *p < 0.01 and **p < 0.03 by Student’s t-test (**C**) and Tukey’s HSD test (**D**). (**E**,**F**) Total insulin content of islets isolated from *db/m* + mice or *db/db* mice administered Ctrl or Ins (n = 4, 5 and 9 islets from the *db/m* + , Ctrl and Ins groups, respectively), or from *db/db* mice administered Ctrl, Img or Met (n = 13, 10 and 11 islets from the Ctrl, Img and Met groups, respectively); *p < 0.01 by Tukey’s HSD test.

We expected that reduction of dysfunctional mitochondria in *db/db* mice would improve β-cell function. Thus, we examined insulin secretion and apoptosis in β-cells from *db/db* mice treated with imeglimin, metformin or insulin. The amount of secreted insulin was dramatically reduced in islets from *db/db* mice, which was significantly ameliorated by insulin treatment (Fig. 5A). Similarly, imeglimin, but not metformin, increased the amount of secreted insulin in *db/db* islets (Fig. 5B). Apoptosis was assessed by TUNEL assay in pancreatic sections, and the number of TUNEL-positive nuclei in pancreatic β-cells was counted. As shown in Fig. 5C,D, the numbers of TUNEL-positive β-cells were markedly reduced in both imeglimin- and insulin-treated *db/db* mice compared with those in control-treated *db/db* mice. Metformin treatment also led to lower levels of apoptotic cell death in these mice, but the results did not reach statistical significance. To further examine whether imeglimin and insulin could suppress apoptosis in β-cells from *db/db* mice, we evaluated the proportion of β-cells of isolated islets by measuring their insulin content. As shown in Fig. 5E, the insulin content of islets from control-treated *db/db* mice was markedly decreased compared with that of *db/m* + mice, but was significantly restored by insulin treatment. Insulin content was also recovered in islets isolated from imeglimin-treated, but not metformin-treated, *db/db* mice compared with that of control-treated *db/db* mice (Fig. 5F).

Taken together, these results suggested that imeglimin treatment reduced dysfunctional mitochondria and ROS production in pancreatic β-cells, which led to the restoration of functional β-cells and amelioration of blood glucose levels in *db/db* mice.

## Discussion

Imeglimin was previously shown to restore mitochondrial morphology^15,16^ and improve mitochondrial function in pancreatic β-cells^20^, suggesting the possibility that imeglimin could also improve mQC. In this study, we administered imeglimin, metformin or insulin to *db/db* mice for 6 weeks and compared their effects in pancreatic β-cells. The accumulation of dysfunctional mitochondria and excessive ROS production in these cells, suggesting the loss of mQC, was adequately restored by treatment with imeglimin or insulin. Furthermore, our results showed that imeglimin and insulin reduced mitophagic activity in β-cells from *db/db* mice. The increased mitophagic activity, accumulation of dysfunctional mitochondria and excessive ROS production in *db/db* β-cells suggested that the generation of dysfunctional mitochondria overwhelmed the degradative capacity of the mitophagy process. Thus, the imeglimin-induced reduction in mitophagic activity in these cells seems to have also reduced the numbers of dysfunctional mitochondria. Taken together with the imeglimin-mediated increase in the amount of secreted insulin and reduction in apoptotic β-cell death, our findings suggest that imeglimin is beneficial for maintaining mQC in diabetic β-cells and preserving β-cell function and euglycemia in T2D patients.

Because ROS can damage mitochondrial components and contribute to the generation of dysfunctional mitochondria^29^, the reduction of ROS would be expected to help improve mQC. In this study, we found that imeglimin and insulin, but not metformin, suppressed ROS production in β-cells from *db/db* mice (Fig. 4), although their mechanisms on the suppression of ROS production would be distinct. Insulin treatment is known to preserve functional β-cells in T2D patients, probably by alleviating the burden of excessive insulin secretion in these cells^24,25^. Because ROS are unavoidable byproducts of mitochondrial respiration^30^, chronic supplementation with exogenous insulin could provide a rest from insulin secretion, thereby suppressing mitochondrial respiration and excessive ROS generation^26^. Meanwhile, both imeglimin and metformin have similar euglycemic effects (Fig. 1), but only imeglimin suppressed ROS production in islet cells from *db/db* mice (Fig. 4). Thus, in contrast to the effect of insulin, the suppression of ROS production by imeglimin might not be related to the reduction in insulin demand caused by the lowering of blood glucose levels; rather, it seems likely that imeglimin reduced ROS production through a direct effect on islet cells. Consistently, ROS generation was decreased in *db/db* islet cells cultured in the presence of imeglimin (Fig. 4).

Imeglimin reportedly upregulates the expression of NAMPT, a key enzyme in the salvage pathway of the NAD^+^ biosynthesis pathway, in pancreatic β-cells^22^. NAMPT converts nicotinamide to nicotinamide mononucleotide, which is then converted to NAD^+^^31^. In cancer cells, NAMPT has been shown to contribute to the cellular capacity to tolerate oxidative stress^31^, most likely through NAD^+^-dependent enzymes, such as those in the sirtuin family. Several sirtuins are expressed in pancreatic β-cells^32^, and sirtuin 3 was notably shown to suppress ROS production and enhance GSIS in these cells^33^. Thus, it seems likely that imeglimin treatment increases the cellular NAD^+^ pool through the upregulation of NAMPT, which might activate sirtuins and reduce ROS generation in pancreatic β-cells. Meanwhile, imeglimin has been reported to reduce ROS generation and inhibit reverse electron transport at complex I in the mitochondrial respiratory chain in human dermal microvascular endothelial cells (HMEC-1) and isolated mitochondria^13,34^. Metformin has also been reported to reduce ROS production by inhibiting reverse electron transport in isolated mitochondria^35^; however, it failed to reduce ROS production in *db/db* β-cells in this study (Fig. 4). Therefore, we speculate that the imeglimin-induced reduction in ROS generation observed in this study was not related to the inhibition of reverse electron transport. Notably, metformin has been reported to suppress ROS production in pancreatic β-cells stimulated with palmitate and high glucose^36^. Therefore, metformin might also inhibit ROS production under certain conditions. Further studies are needed to elucidate the mechanism by which imeglimin suppresses ROS generation and improves mQC in pancreatic β-cells.

We observed that imeglimin, but not metformin, improved GSIS and suppressed apoptosis in pancreatic β-cells (Fig. 5). Because ROS were previously shown to disrupt GSIS and induce apoptosis^30^, it follows that improved β-cell function under imeglimin treatment might be mediated by reduced ROS production and the restoration of mQC. Imeglimin has also been shown to alleviate endoplasmic reticulum stress, leading to the suppression of β-cell apoptosis^20^. Furthermore, the imeglimin-mediated activation of the NAD^+^–sirtuin axis described above may enhance GSIS and inhibit apoptosis in *db/db* β-cells. Hallakou-Bozec et al. showed that an increase in NAD^+^ enhanced the mobilization of Ca^2+^ through the NAD^+^–cyclic ADP ribose–ryanodine receptor axis, resulting in augmentation of GSIS^22^. NAD^+^-induced sirtuin activation was also shown to augment GSIS and suppress apoptotic β-cell death^32,33,37,38^. Continued elucidation of the effects of increasing the cellular NAD^+^ pool on GSIS and apoptosis through mechanisms involving sirtuins would contribute to a deeper understanding of the action of imeglimin.

In summary, we demonstrated that imeglimin, but not metformin, mitigated the accumulation of dysfunctional mitochondria and led to an increase in the amount of secreted insulin and suppression of apoptosis in pancreatic β-cells from *db/db* mice. Our findings suggest that maintenance of mQC is important for preserving β-cell function and survival, and support the use of imeglimin treatment in T2D patients, especially for the preservation of β-cell function.

## Methods

### Animals

Male mice were used in all of the experiments in this study. All mice were housed under a 12 h light/12 h dark cycle in climate-controlled facilities. Animal experiments were approved by the Kyorin University Animal Care Committee (Permission no. 238), and were conducted in accordance with the relevant guidelines and regulations of Kyorin University and ARRIVE guidelines (https://arriveguidelines.org). *BKS.Cg-+Lept*^*db*^*/+Lept*^*db*^*/Jcl* (*db/db*) and *BKS.Cg-m+/+Lept*^*db*^*/jcl* (*db/m* +) mice were purchased from CLEA Japan (Tokyo, Japan). The *db/m* + mice were crossed with *CMMR*^*flox*^*;RIP-Cre* mice^26^ to obtain *db/m* +*;CMMR*^*flox*^*;RIP-cre* mice, which were then crossed for more than five generations into the C57BLKS/J (BKS) background. Finally, *db/m* + *;CMMR*^*flox/flox*^*;RIP-cre* female mice were crossed with *db/m* + male mice to generate *db/db;CMMR*^*flox*^*;RIP-cre* mice. We divided *db/db* mice into three groups and started interventions at the age of 10 weeks. The mice were orally administered 200 mg/kg body weight of imeglimin (provided by Sumitomo Pharma Co. Ltd., Japan) or distilled water (control) twice a day, or 300 mg/kg metformin (Fujifilm-Wako, Japan) once a day, for 6 weeks. For chronic insulin treatment, insulin (36 μg/day; Fujifilm-Wako) or volume-matched saline (control) was infused continuously using an osmotic minipump (ALZET, USA) for 6 weeks^26^. Mice were fasted for 18 h or 4 h before being subjected to an oral glucose tolerance test using 0.8 g glucose/kg body weight or an insulin tolerance test using 1.5 U/kg Humulin R (Eli Lilly, USA), respectively. Blood samples were collected from the tail vein, and blood glucose concentrations and serum insulin were measured using a GlutestR (Sanwa Kagaku Kenkyusho Co., Japan) or an insulin ELISA kit (Morinaga, Japan). Pancreatic islets of Langerhans cells were prepared as described previously^11,39^.

### Imaging analysis

Mitophagy signal measurements of CMMR mouse pancreatic sections, ROS measurements using MitoSOX, functional mitochondrial imaging using MitoTracker Orange CM-H_2_TMRos (Thermo Fisher Scientific) and immunostaining were performed as described previously^26^. To detect apoptotic death of β-cells, pancreatic sections were labeled with a MEBSTAIN Apoptosis TUNEL kit (MBL, Japan) in accordance with the manufacturer’s instructions, followed by staining with anti-insulin antibody (Sigma) and 4′,6-diamidino-2-phenylindole (DAPI). The numbers of TUNEL-labeled nuclei among insulin-positive β-cells were manually counted.

### Immunoblotting, and insulin secretion and Oxyblot assays

Insulin secretion assays and immunoblotting were performed as described previously^26^. OxyBlot assays were performed in accordance with the manufacturer’s instructions (Merck, Germany). Antibodies against BNIP3 (Cell Signaling Technology, #3769S) and α-tubulin (Sigma, #T9026) were purchased from commercial sources. Membranes were cut prior to antibody application. Specific detections of the target antigens by these antibodies were confirmed using mouse islet samples (Supplemental Figs. S5, S6).

### Statistical analysis

Data are expressed as the mean ± standard error of the mean. Statistical analysis was performed using the scipy.stats (https://scipy.org) and statsmodels.stats (https://www.statsmodels.org) libraries. The equality of variance and normality were tested in all experiments. The significance of differences between datasets was assessed using statistical tests, as indicated in each figure legend. A p value less than 0.05 was considered to indicate statistical significance.

### Supplementary Information

Supplementary Figures.

## Data Availability

Datasets that were generated and/or analyzed during this study are available from the corresponding author upon reasonable request.
