# Supplementary material for: Imeglimin mitigates the accumulation of dysfunctional mitochondria to restore insulin secretion and suppress apoptosis of pancreatic β-cells from db/db mice
Source: Sci Rep. 2024 Mar 14;14:6178. doi: 10.1038/s41598-024-56769-w (PMC10940628; doi:10.1038/s41598-024-56769-w)
Supplement: Supplementary file 1 — Supplementary Figures. [file 41598_2024_56769_MOESM1_ESM.pdf]

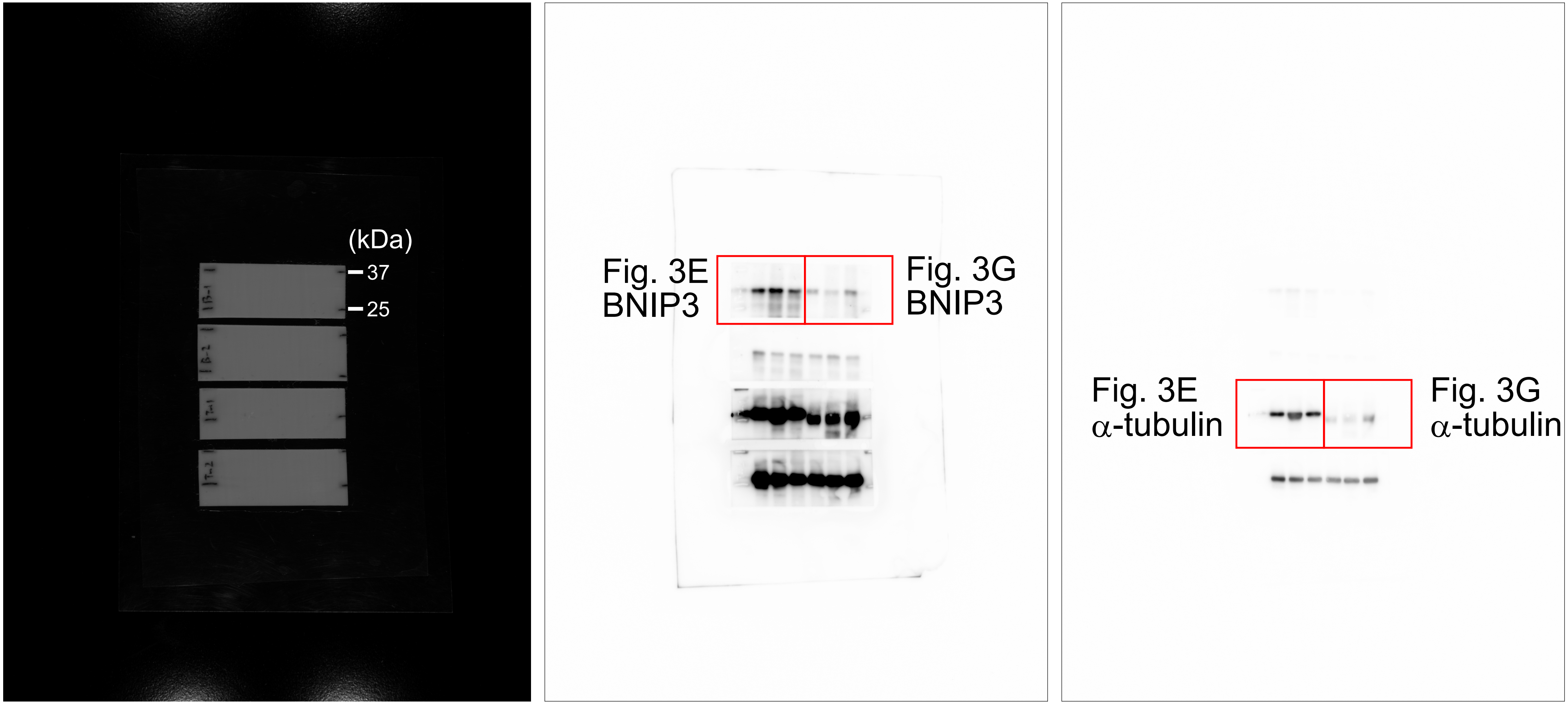

Supplemental Figure 1
Original membrane and immunoblot images supplemental to Fig. 3E, 3G

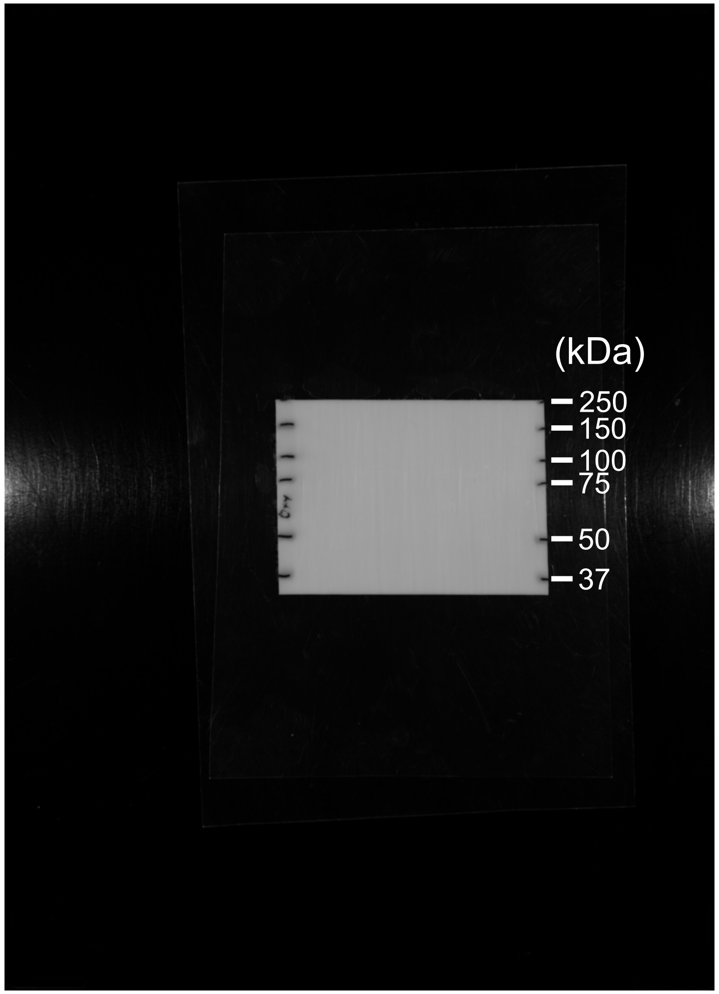

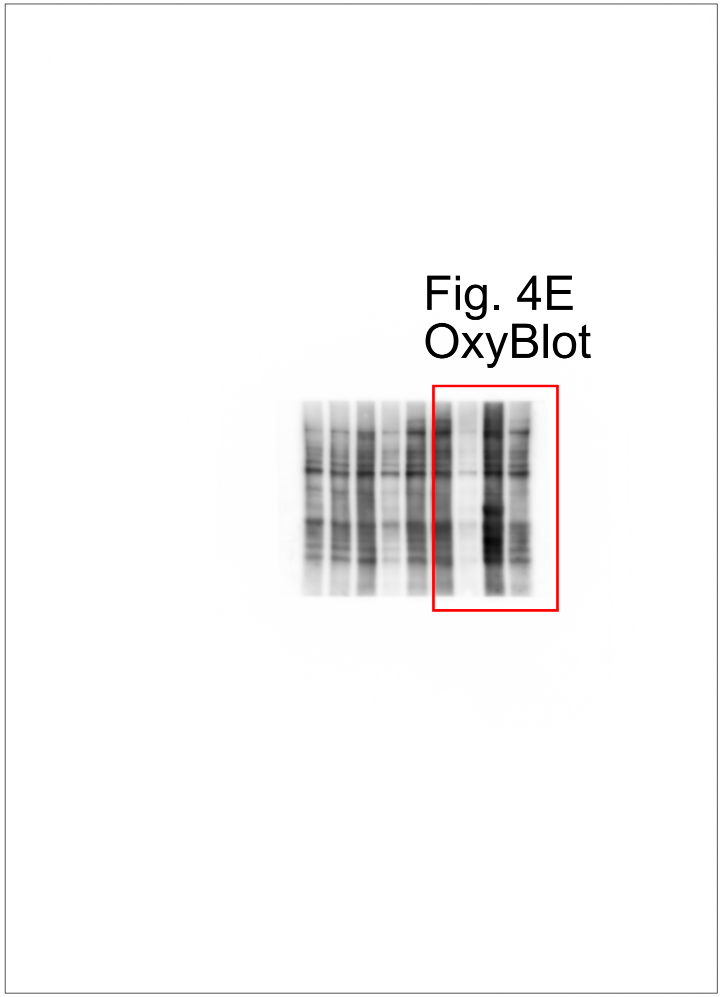

Supplemental Figure 2
Original membrane and immunoblot images supplemental to Fig. 4E

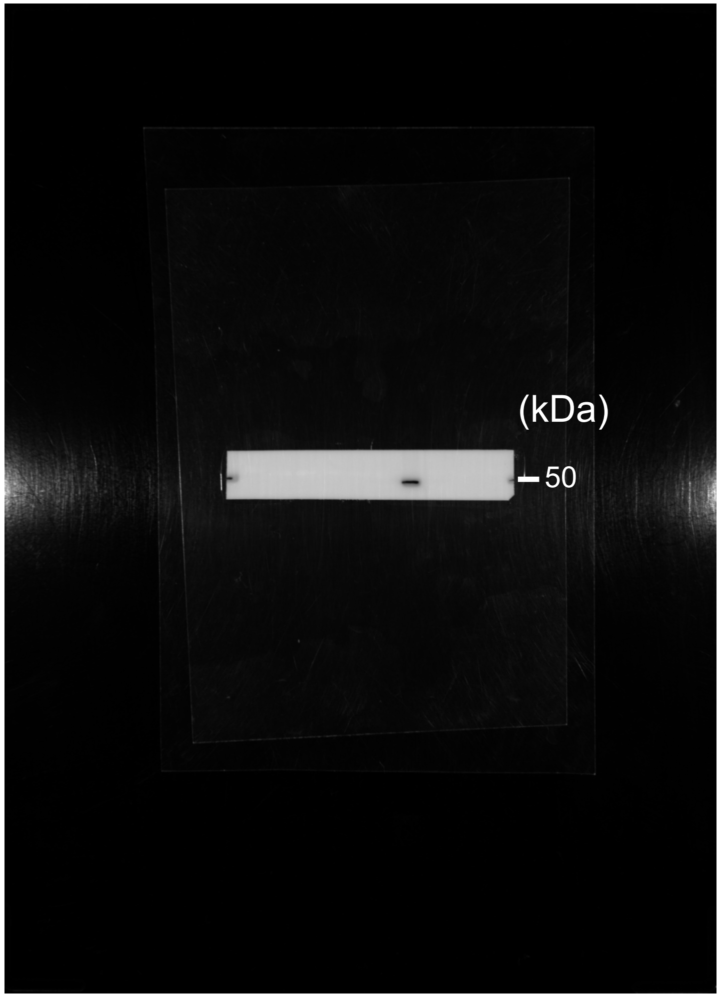

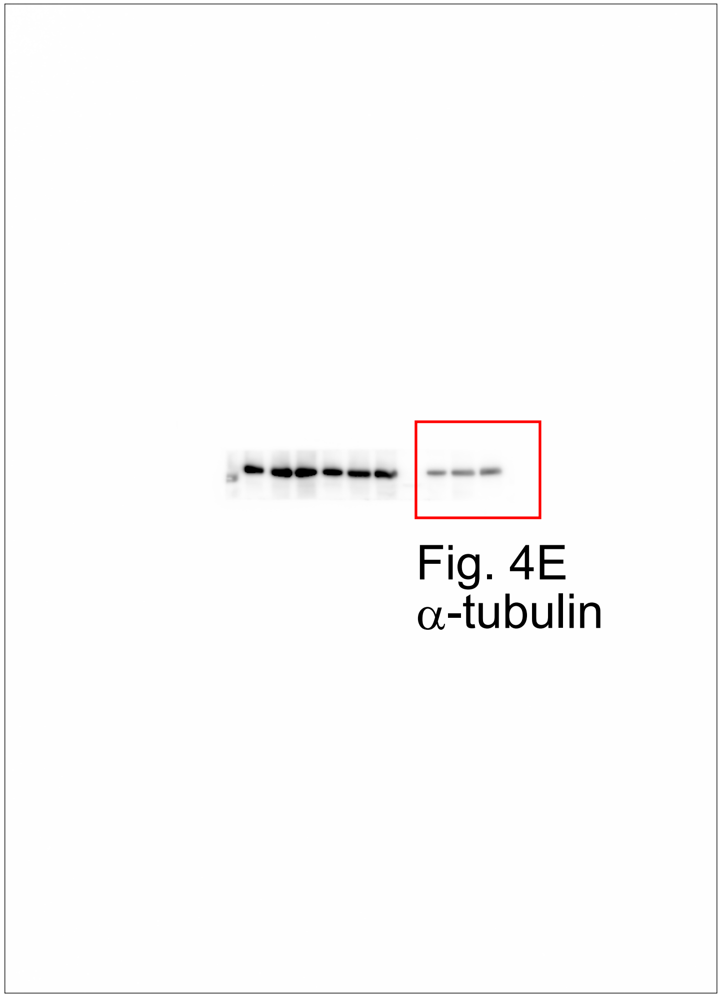

Supplemental Figure 3
Original membrane and immunoblot images supplemental to Fig. 4E

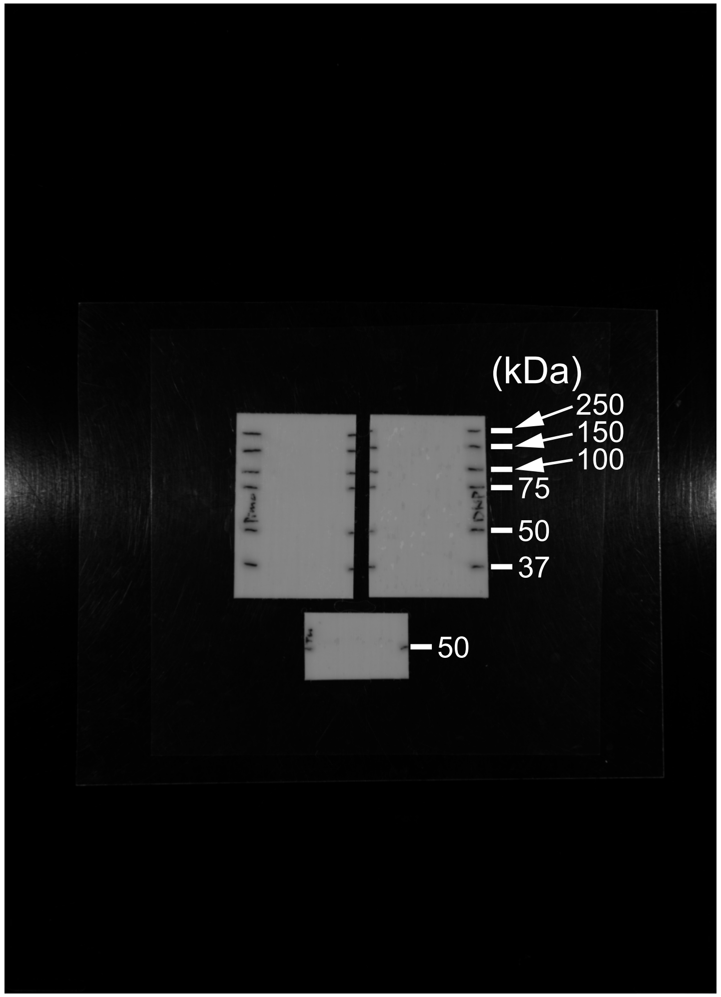

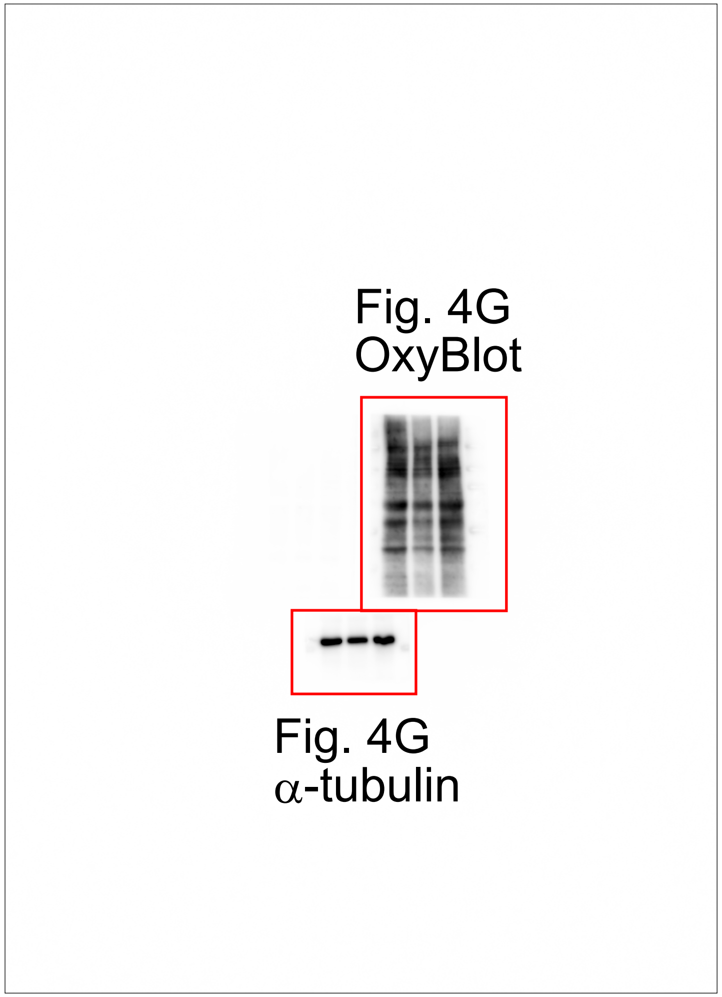

Supplemental Figure 4
Original membrane and immunoblot images supplemental to Fig. 4G

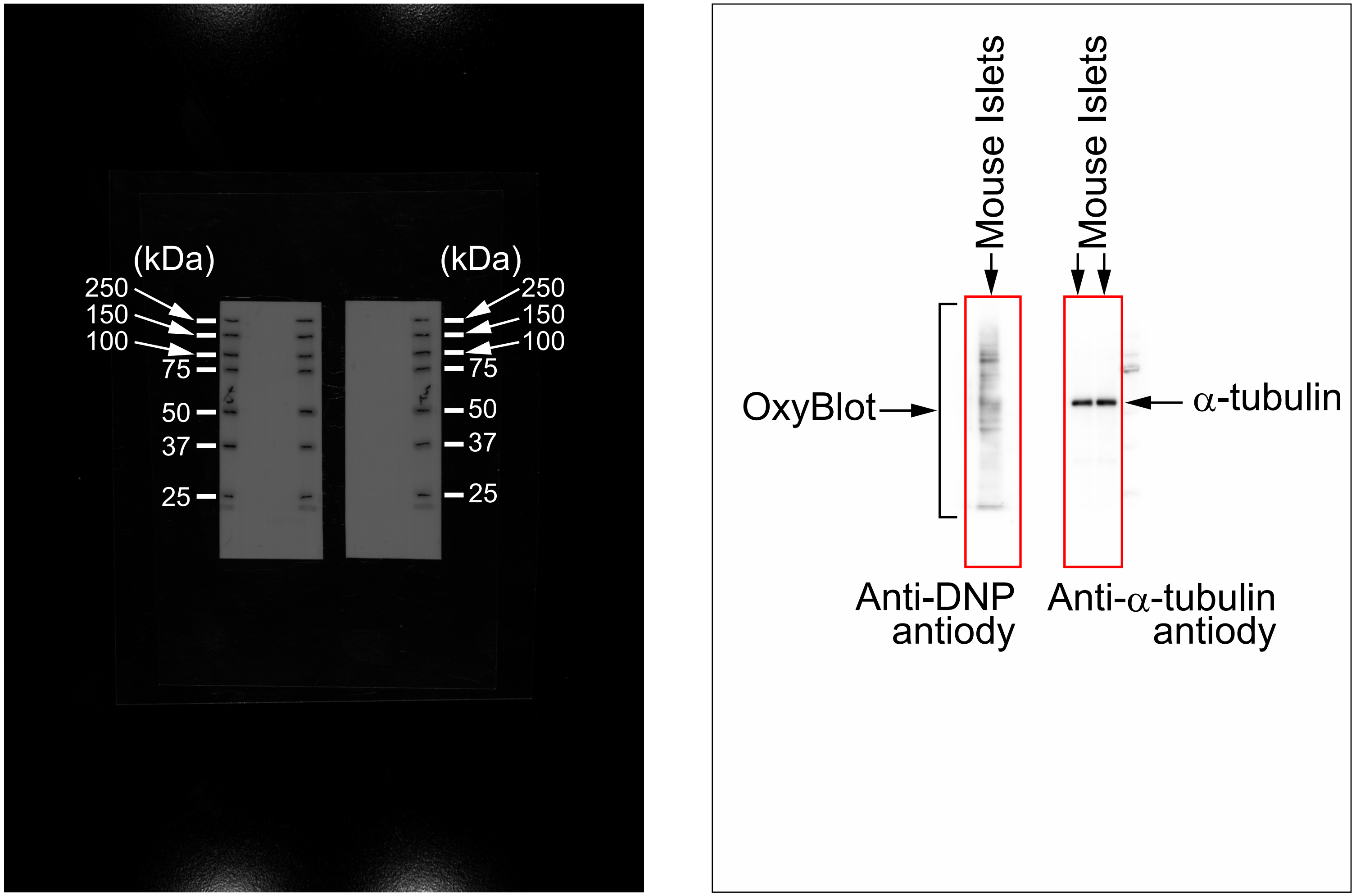

Supplemental Figure 5
Uncropped membrane and immunoblot images for anti-DNP antibody (OxyBlot) and anti-α-tubulin antibody

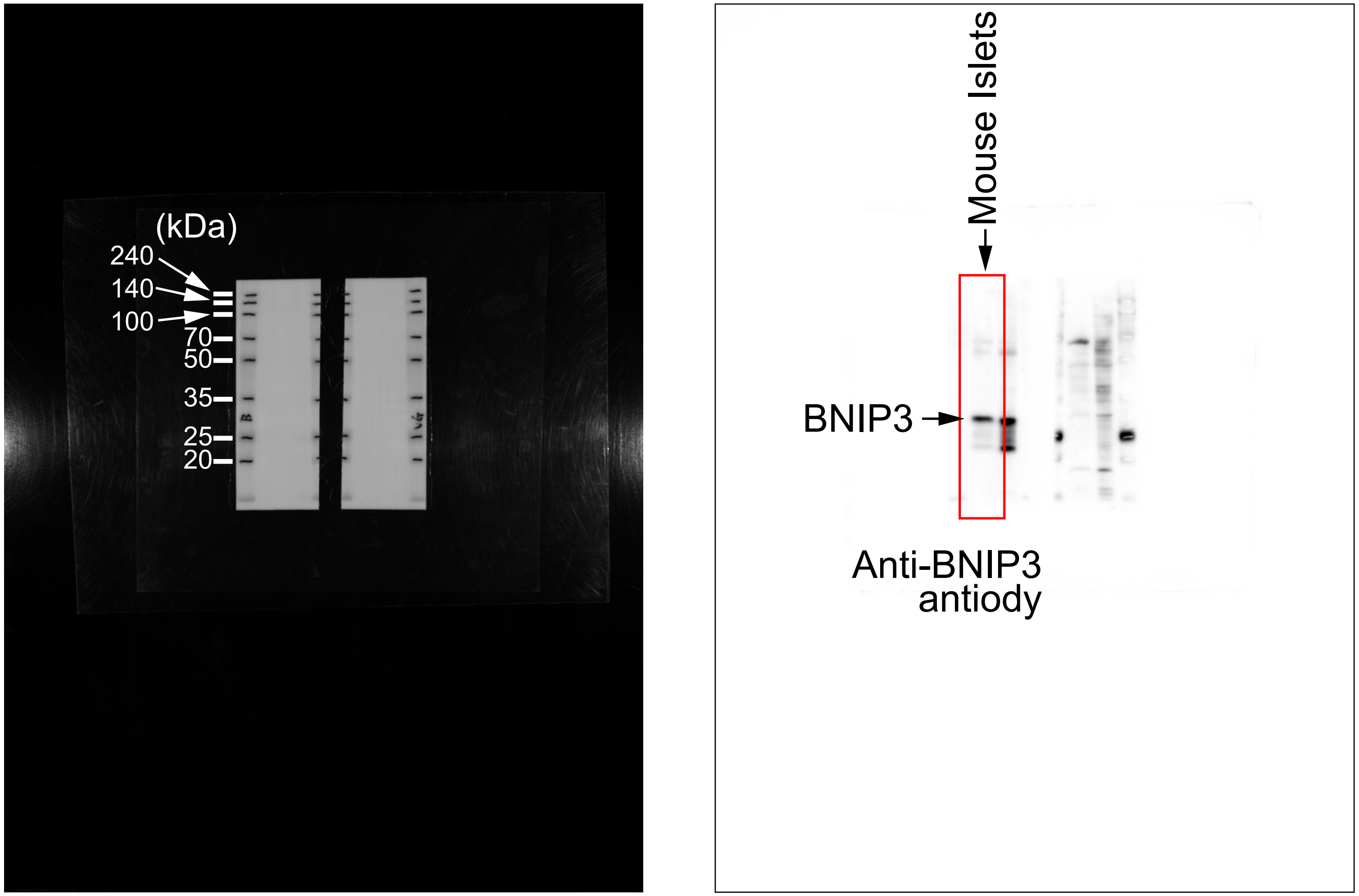

Supplemental Figure 6
Uncropped membrane and immunoblot images for anti-BNIP3 antibody
